# Construction of a circular RNA–microRNA–messenger RNA regulatory network of hsa_circ_0043256 in lung cancer by integrated analysis

**DOI:** 10.1111/1759-7714.14226

**Published:** 2021-11-21

**Authors:** Yongwen Li, Ruifeng Shi, Guangsheng Zhu, Chen Chen, Hua Huang, Min Gao, Songlin Xu, Peijun Cao, Zihe Zhang, Di Wu, Xuanguang Li, Hongyu Liu, Jun Chen

**Affiliations:** ^1^ SynBio Research Platform, Collaborative Innovation Center of Chemical Science and Engineering (Tianjin), School of Chemical Engineering and Technology Tianjin University Tianjin China; ^2^ Tianjin Key Laboratory of Lung Cancer Metastasis and Tumor Microenvironment, Tianjin Lung Cancer Institute Tianjin Medical University General Hospital Tianjin China; ^3^ Department of Lung Cancer Surgery Tianjin Medical University General Hospital Tianjin China; ^4^ Department of Thoracic Surgery The Affiliated Hospital of Inner Mongolia Medical University Huhhot China; ^5^ Quantitative Biomedical Research Center, Department of Population and Data Sciences University of Texas Southwestern Medical Center Dallas Texas USA; ^6^ Department of Thoracic Surgery, First Affiliated Hospital, School of Medicine Shihezi University Shihezi China

**Keywords:** bioinformatics analysis, circRNA, hsa_circ_0043256, lung cancer, regulatory network

## Abstract

**Background:**

Patients with non‐small cell lung cancer (NSCLC) are diagnosed in advanced stages and with a poor 5‐year survival rate. There is a critical need to identify novel biomarkers to improve the therapy and overall prognosis of this disease.

**Methods:**

Differentially expressed genes (DEGs) were identified from three profiles of GSE101586, GSE101684 and GSE112214 using Venn diagrams. hsa_circ_0043256 were validated using quantitative real‐time polymerase chain reaction (RT‐qPCR). The circular RNA–microRNA–messenger RNA (circRNA–miRNA–mRNA) regulatory network was constructed with Cytoscape 3.7.0. Hub genes were identified with protein interaction (PPI) and validated with the Gene Expression Profiling Interactive Analysis (GEPIA), Human Protein Atlas (HPA) databases, and immunohistochemistry. Survival analyses were also performed using a Kaplan–Meier (KM) plotter. The effects of hsa_circ_0043256 on cell proliferation and cell cycles were evaluated by EdU staining and flow cytometry, respectively.

**Results:**

hsa_circ_0043256, hsa_circ_0029426 and hsa_circ_0049271 were obtained. Following RT‐qPCR validation, hsa_circ_0043256 was selected for further analysis. In addition, functional experiment results indicated that hsa_circ_0043256 could inhibit cell proliferation and cell‐cycle progression of NSCLC cells in vitro. Prediction by three online databases and combining with DEGs identified from The Cancer Genome Atlas (TCGA), a network containing one circRNAs, three miRNAs, and 209 mRNAs was developed. Gene Ontology (GO) and Kyoto Encyclopedia of Genes and Genomes (KEGG) pathway analysis indicated DEGs might be associated with lung cancer onset and progression. A PPI network based on the 209 genes was established, and five hub genes (BIRC5, SHCBP1, CCNA2, SKA3, and GINS1) were determined. Following verification of five hub genes using GEPIA database, HPA database, and immunohistochemistry. High expression of all five hub genes led to poor overall survival.

**Conclusion:**

Our study constructed a circRNA–miRNA–mRNA network of hsa_circ_0043256. hsa_circ_0043256 may be a potential therapeutic target for lung cancer.

## Graphical abstract

Flowchart of the study. First, three datasets from GEO to obtain DECs, and validated by RT‐qPCR and functional experiments in vitro. Next, the prediction miRNAs and differentially expressed miRNAs obtained from TCGA were intersected. We then constructed a circRNA–miRNA–mRNA network around hsa_circ_0043256 and GO and KEGG analyses. Subsequently, a PPI network was established and hub genes were identified and validated with GEPIA, HPA databases, immunohistochemistry, and a KM plotter.

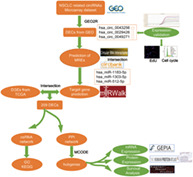

## INTRODUCTION

Lung cancer is the leading cause of cancer‐related death worldwide.^1^ Although much progress has been achieved in the past 10 years in treatment of lung cancer, only 16.8% of all lung cancer patients survive for 5 years or more after diagnosis.^2^, ^3^Therefore, there is an urgent need to better understand the mechanism of lung cancer development to identify screening novel biomarkers and develop therapeutic approaches to improve and overall prognosis of this disease.

Circular RNA (circRNA) is a newly discovered type of non‐coding RNA (ncRNA) and ubiquitously exists in many species.^4^ An increasing number of studies demonstrate that circRNAs can function as molecular sponges for (microRNA) miRNAs and RNA‐binding proteins (RBPs) and play important roles in various biological processes, including as tumor suppressors or proto‐oncogenes in the occurrence and development of cancers.^4^ In lung cancer, circRNAs also play a crucial role in many physiological processes.^5^ For instance, circTP63 influences cell‐cycle progression by interacting with the miR‐873‐3p and prevents miR‐873‐3p contributing to decreased levels of FOXM1 in lung squamous cell carcinoma.^6^ However, the functions of circRNAs and their related molecular mechanisms in lung cancer remain largely unknown.

**FIGURE 1:**
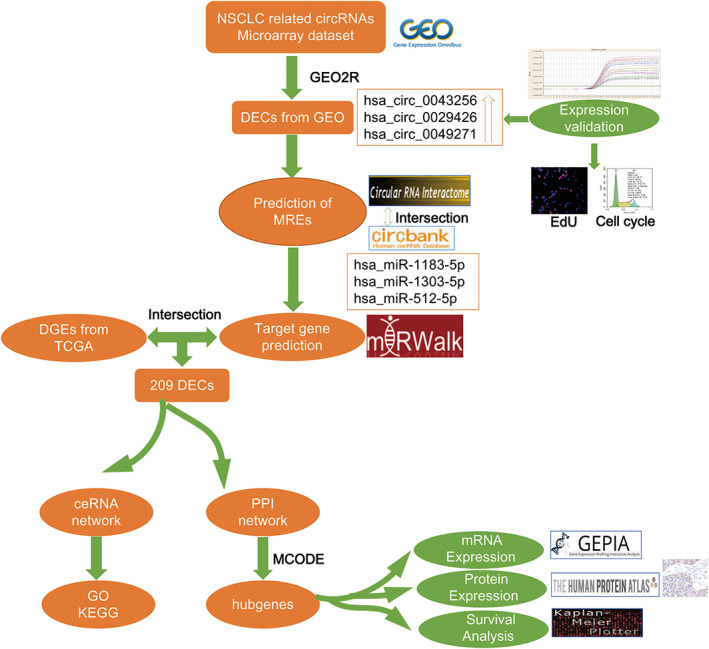
Flowchart of the study. First, three datasets from GEO to obtain DECs, and validated by RT‐qPCR and functional experiments in vitro. Next, the prediction miRNAs and differentially expressed miRNAs obtained from TCGA were intersected. Next, we constructed a circRNA–miRNA–mRNA network around hsa_circ_0043256 and GO and KEGG analyses. Subsequently, a PPI network was established and hub genes were identified, and validated with GEPIA, HPA databases, and immunohistochemistry, and a KM plotter

In this work, we have downloaded three lung cancer‐associated circRNA expression profiles (GSE101586, GSE101684, and GSE112214) from Gene Expression Omnibus (GEO) database, and performed an integrated analysis of circRNAs, miRNAs, and (messenger RNAs) mRNAs expression profiles. The flowchart for this procedure is shown in Figure 1. The differentially expressed mRNAs (DEGs), differentially expressed miRNAs (DEIs), and differentially expressed circRNAs (DECs) were identified. Importantly, the expression of hsa_circ_0043256 and its biological functions were validated in vitro, and ceRNA (circRNA–miRNA–mRNA) regulatory network was conducted. This study will illuminate the underlying mechanisms of lung cancer and may help provide reliable biomarkers for early diagnosis of this disease.

## MATERIALS AND METHODS

### Data collection

Three circRNA expression profiles (GSE101586, GSE101684 and GSE112214) were downloaded from the GEO, (www.ncbi.nlm.nih.gov/geo/), analyzed on the Agilent‐069978 Arraystar Human CircRNA microarray V1 GPL19978 platform. Lung cancer associated mRNA sequencing data were downloaded from The Cancer Genome Atlas (TCGA) (https://cancergenome.nih.gov/) on January 8, 2020. A total of 1145 samples were included in this study, containing 1016 lung cancer samples and 129 matched normal samples.

#### Patients and samples

Twelve non‐small cell lung cancer (NSCLC) patients who were diagnosis with NSCLC and underwent surgical resection of lung cancer between 2015 and 2020 at the Department of Lung Cancer Surgery, Tianjin Medical University General Hospital were included. Written informed consent was obtained from each participant. The clinical and pathological characteristics of 12 NSCLC patients are presented in Table S1. The study was conducted in accordance with the Declaration of Helsinki (as revised in 2013) and approved by the Review Board of Tianjin Medical University General Hospital .

**TABLE 1:** Essential characteristics of the three DECs

CircRNA ID	Up/down	Position	Strand	Length	Best transcript	Gene symbol
hsa_circ_0043256	Down	Chr17:35604934–35609962	−	483	NM_198839	ACACA
hsa_circ_0029426	Down	Chr12:131357380–131357465	+	85	NM_006325	RAN
hsa_circ_0049271	Down	Chr19:10610070–10610756	−	686	NM_203500	KEAP1

### Differential expression analysis of circRNAs

The raw data of microarray datasets was preprocessed using GEO2R. The criteria for selection of DECs were *p*‐value or adj. *p*‐value <0.05 and |log2FC| >1. CircRNAs with statistical significance between lung cancer and non‐tumor tissues in GSE101586, GSE101684, and GSE112214 were screened, respectively. Next, circRNAs upregulated or downregulated in both profiles were selected and identified. The DECs intersection of three datasets was analyzed using a Venn diagram (http://bioinformatics.psb.ugent.be/webtools/Venn/). The structure of circRNAs and the secondary stem‐loop structure of miRNAs were also predicted using the cancer‐specific circRNA database (CSCD).^7^

### Construction of circRNA–miRNA‐mRNA regulatory network

To predict the interactions of circRNA and miRNA, circular RNA Interactome^8^ (https://circinteractome.nia.nih.gov) and circBank (http://www.circbank.cn/) were used. We identified overlapped miRNAs of the two algorithms as potential target miRNAs (DEIs) of the DECs. In addition, we used miRWalk (version 2.0; http://zmf.umm.uni-heidelberg.de/),^9^of which four bioinformatic algorithms (RNA22, miRanda, miRWalk, and Targetscan) were selected to predict miRNA‐targeted mRNAs of the three essential miRNAs. Only mRNAs recognized by all the four algorithms were considered to be candidate mRNAs. All the candidate mRNAs predicted to the three essential miRNAs were merged with the differentially expressed genes (DEGs) identified from TCGA between lung cancer and non‐tumor tissues. The common DEGs across miRNA‐targets and the TCGA were analyzed with Venn plot. The circRNA–miRNA–mRNA regulatory network was constructed using Cytoscape 3.7.0 software^10^ using the correlations between DECs, DEIs, and DEGs.

### Bioinformatic DEG analysis

Gene Ontology (GO) and Kyoto Encyclopedia of Gene and Genomes (KEGG) enrichment analyses were performed with the Database for Annotation, Visualization and Integrated Discovery (DAVID) using the GO and KEGG pathway methods. The results were downloaded and visualized using the Goplot package. A GO term or KEGG pathway with adj. *p*‐value < 0.05 was considered to indicate a statistically significant difference. The top 20 enriched GO terms and pathways of DEGs were ranked according to enrichment score (log10 [*p* value]). The PPI network of 209 DEGs was established by the STRING 10.5 and visualized by the Cytoscape 3.7.1.

### Reconstruction of the circRNA–miRNA–hub gene network

Hub‐genes were selected by Cytoscape Molecular Complex Detection (MCODE) app from the PPI network. To identify the association between DECs, DEIs, and hub genes, the subnetwork of circRNA–miRNA–hub gene network was identified.

### Validation of hub genes

The Gene Expression Profiling Interactive Analysis (GEPIA) (http://gepia.cancer-pku.cn/) was used to compare differential expression of common cancer types and their normal adjacent tissues. The Human Protein Atlas (HPA) (http://www.proteinatlas.org/) was used to validate real hub gene. Kaplan–Meier (KM) plotter (http://kmplot.com/analysis/index.php) database was applied for survival analyses of hub‐genes.

### Cell culture and transient transfection

The human lung adenocarcinoma (LUAD) cell lines HCC827, H1650, and H2291 cells were purchased from the American Type Culture Collection (Manassas, VA). Other human NSCLC cell lines (H1975, H1299, H3255, 95D, and PC‐9) and the normal human bronchial epithelial cell line BEAS‐2B were obtained from Cell Bank of the Chinese Academy of Sciences (Shanghai, China). All cell lines were cultured in RPMI 1640 medium containing 10% fetal bovine serum (Gibco, Carlsbad, CA). Human hsa_circ_0043256 overexpression plasmid and the negative control were generated by GeneChem (GeneChem, China). For transient transfection, cells were seeded in six‐well culture plates, and transfection was performed at 90% cell confluence using Lipofectamine 3000 (Invitrogen, Carlsbad, CA) according to the manufacturer's instructions.

### RNA extraction, real‐time polymerase chain reaction, and quantitative real‐time polymerase chain reaction

Total RNA was extracted from cultured cells or tissues using TRIzol Reagent (Invitrogen, Carlsbad, CA) and reverse transcribed with PrimeScript RT reagent Kit (TaKaRa) according to the manufacturer's instructions. Quantitative real‐time polymerase chain reaction (qRT‐PCR) analyses were performed with Hieff qPCR SYBR Green Master Mix (Yeasen Biotechnology, Shanghai).The results were normalized with respect to the expression of GAPDH. The details of the primers used are as follows: GAPDH, Forward (F): 5′‐GGAGCGAGATCCCTCCAAAAT‐3′, Reverse (R): 5′‐GGCTGTTGTCATACTTCTCATGG‐3′; hsa_circ_0029426, Forward (F): 5′‐TGAAACGTCATTTGACTGGTG‐3′, Reverse (R) 5′‐CCAGTACCACCATCAC CAAC‐3′; hsa_circ_0043256, Forward (F): 5′‐AGTGACGGTGGACTGCTCTT‐3′, Reverse (R) 5′‐TCGAAAGTCACCCCG AATAG‐3′; hsa_circ_0049271, Forward (F): 5′‐AACTTCGCTGAGCAGATTGG‐3′, Reverse (R) 5′‐GCATGGGGTTCCAGAAG ATA‐3′.

### EdU staining and cell‐cycle analysis

For EdU staining, EdU reagent (RiboBio, Guangzhou, China) was added to each well. After 2 hours, the cells were fixed with 4% formaldehyde for 15 minutes and treated with 0.5% Triton X‐100 for permeabilization. Next, Apollo reaction cocktail was added to each well, followed by cell staining with Hoechst 33342. For cell‐cycle analyses, cells were harvested and fixed overnight with at 4°C in 70% ethanol. Thereafter, the cells were washed twice with phosphate‐buffered saline (PBS) and then stained with the PI/RNase staining buffer (BD Biosciences, USA). Cell‐cycle distribution was determined using flow cytometry with ModFit software. Each experiment was repeated three times.

### Immunohistochemistry

For immunohistochemistry (IHC), sections were washed in PBS and blocked with 5% bovine serum albumin for 15 minutes at room temperature. The sections were then incubated with the primary antibody against BIRC5 (1:250) (Abcam, USA), SHCBP1 (1:200) (Bioss, China), CCNA2 (1:100) (Abcepta, China), SKA3 (1:200) (Bioss, China), or GINS1 (1:200) (CUSABIO Technology, China) at 4°C overnight.

### Statistical analysis

Statistical analysis was performed using SPSS (version 23.0, Chicago, IL) and GraphPad Prism 7 (GraphPad Software, La Jolla, CA) software. Student's *t*‐tests were used for comparison of two sample groups. Differences were considered as statistically significant when *p* < 0.05.

## RESULTS

### Identification of DECs and DEGs in lung cancer

**FIGURE 2:**
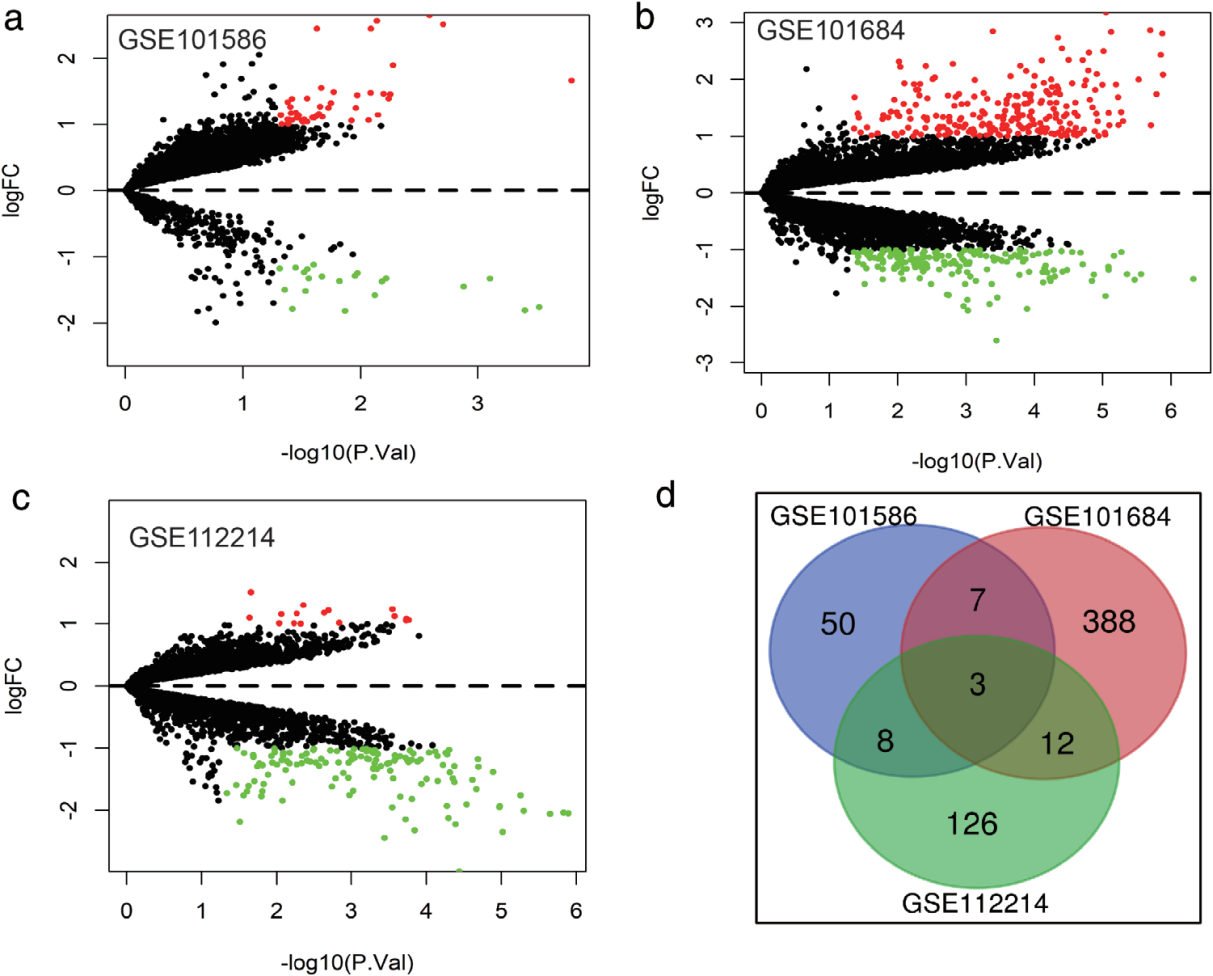
Volcano plots for DECs in NSCLC based on the three microarray datasets from GEO. (a) GSE101586. (b) GSE101684. (c) GSE112214. (d) The three circRNAs that were identified by intersection of circRNAs from the three datasheets

**FIGURE 3:**
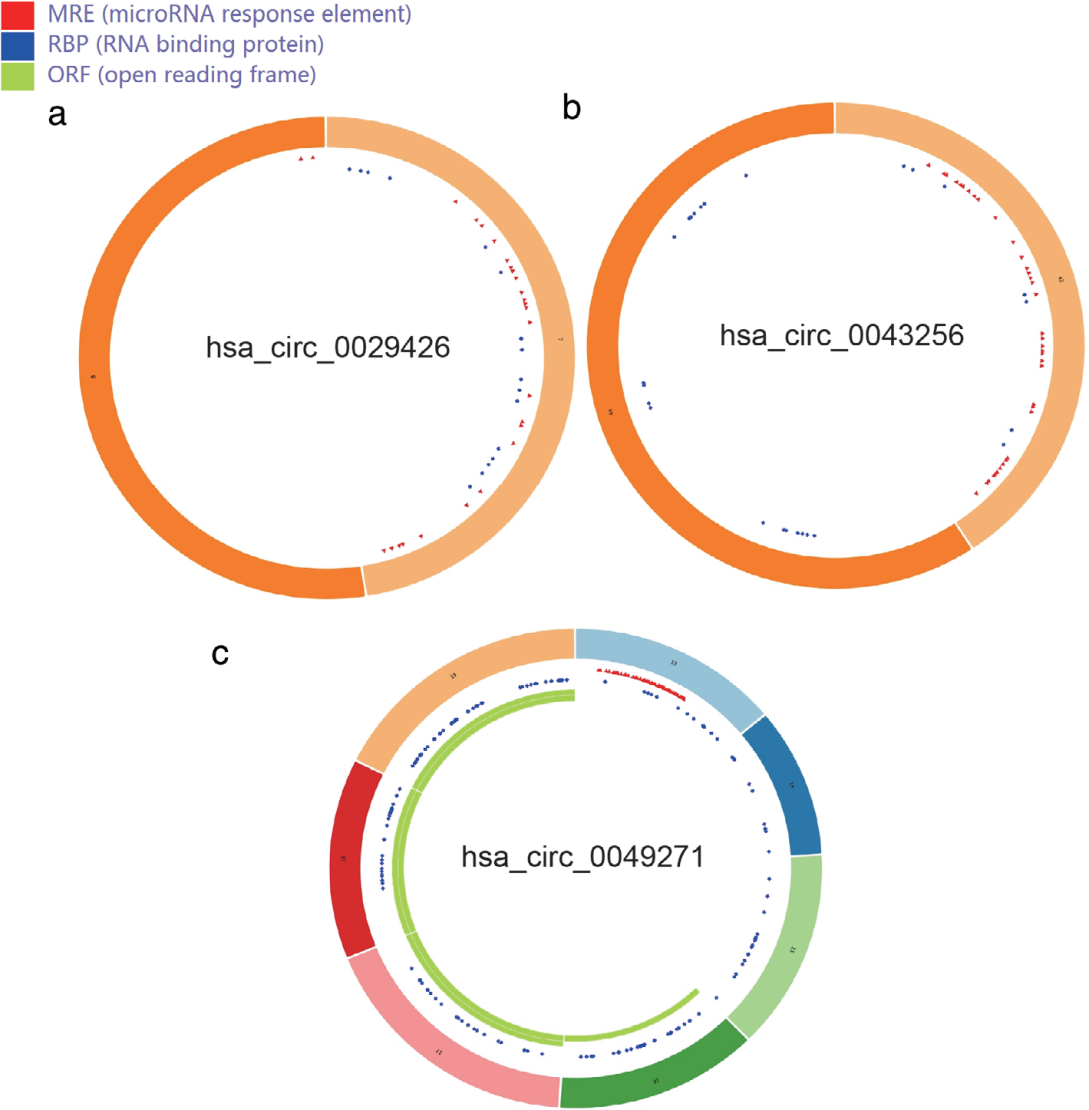
Structural patterns of the three circRNAs by CSCD. (a) hsa_circ_0029426. (b) hsa_circ_0043256. (c) hsa_circ_0049271

Three microarray datasets (GSE101586, GSE101684, and GSE112214) were included in this study, the basic information of which are shown in Table 1. A total of 68 DECs with 47 upregulated circRNAs and 21 downregulated circRNAs were identified from GSE78520 (Figure 2(a)); 410 DECs with 236 upregulated circRNAs and 174 downregulated circRNAs were identified from GSE101684 (Figure 2(b)); 149 DECs with 16 upregulated circRNAs and 133 downregulated circRNAs were identified from GSE112214 (Figure 2(c)). A total of three downregulated circRNAs (hsa_circ_0043256, hsa_circ_0029426, and hsa_circ_0049271) were obtained using a Venn diagram of three datasheets (Table 1). The essential characteristics of the three DECs are displayed in Table 1. The basic structural patterns of the three circRNAs are exhibited in Figure 3.

### Validation of DECs in NSCLC tissues and cell lines

**FIGURE 4:**
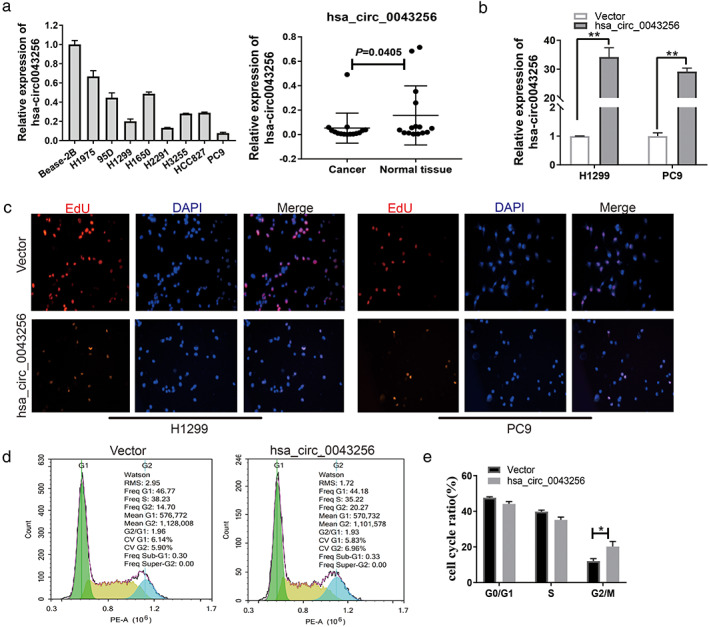
hsa_circ_0043256 inhibits cell proliferation and cell‐cycle progression of NSCLC cells in vitro. (a) hsa_circ_0043256 mRNA levels in cell lines and tissues were measured by RT‐qPCR. (b) hsa_circ_0043256 mRNA levels in H1299 and PC9 cells transfected with hsa_circ_0043256 overexpression plasmid. (c) Representative images of vector‐, and hsa_circ_0043256 plasmid‐ transfected H1299 and PC9 cells analyzed by EdU staining assay. (d), (e) Representative images and quantification of vector‐, and hsa_circ_0043256 plasmid‐ transfected H1299 and PC9 cells analyzed using cell‐cycle assay. *p < 0.05, **p < 0.01. All data are represented by mean ± SD

**FIGURE 5:**
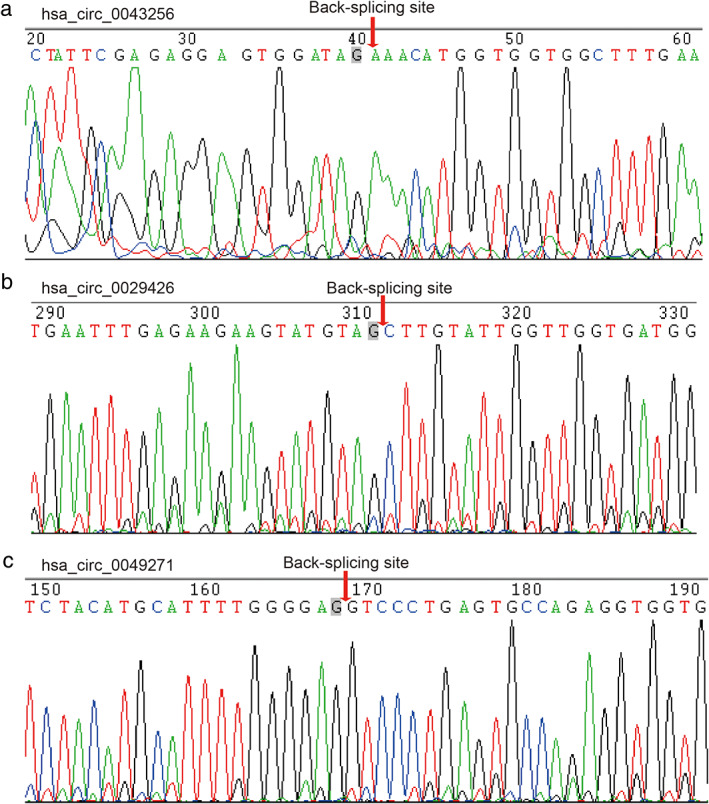
Head‐to‐tail splicing in the RT‐qPCR product of three circRNAs by sanger sequencing. (a) hsa_circ_0029426. (b) hsa_circ_0043256. (c) hsa_circ_0049271

qPCR assay was used to test the expression of three selected circRNAs in 12 pairs of lung cancer samples and adjacent non‐cancerous tissues. The results were verified using Sanger sequencing. The results of qPCR showed that hsa_circ_0043256 was significantly downregulated in lung cancer patient tissues (Figure 4(a)) (*p* = 0.0405), whereas the expression of hsa_circ_0029426 and hsa_circ_0049271 in lung cancer tissues was similar to that observed for non‐cancerous tissues (Figure S1). Furthermore, the head‐to‐tail splicing in the qRT‐PCR product of hsa_circ_0043256, hsa_circ_0029426, and hsa_circ_0049271 was confirmed by Sanger sequencing (Figure 5). Therefore, hsa_circ_0043256 was selected for follow‐up analysis.

### hsa_circ_0043256 promotes NSCLC cell proliferation and controls cell‐cycle progression in vitro

The potential roles of hsa_circ_0043256 in NSCLC biology remain unclear. The expression of hsa_circ_0043256 was determined in eight lung cancer cell lines and the normal human bronchial epithelial cell line BEAS‐2B was determined using qRT‐PCR. Results showed that hsa_circ_0043256 was significantly downregulated in lung cancer cell lines (Figure 4(a)). We performed EdU staining and flow cytometry to investigate the effect of hsa_circ_0043256 on the cell proliferation of the NSCLC cells. H1299 and PC9 cell lines were transfected with hsa_circ_0043256 overexpression plasmid and the negative control (Figure 4(b)). The results of EdU staining demonstrated that overexpression of hsa_circ_0043256 can inhibit proliferation of NSCLC cells (Figure 4(c)). Furthermore, flow cytometry showed that hsa_circ_0043256 can also inhibit the progression of the cell cycle of NSCLC cells (Figure 4(d)). Taken together, hsa_circ_0043256 was capable of inhibiting cell proliferation and cell‐cycle progression of NSCLC cells in vitro.

### Construction of the circRNA–miRNA‐mRNA network

**FIGURE 6:**
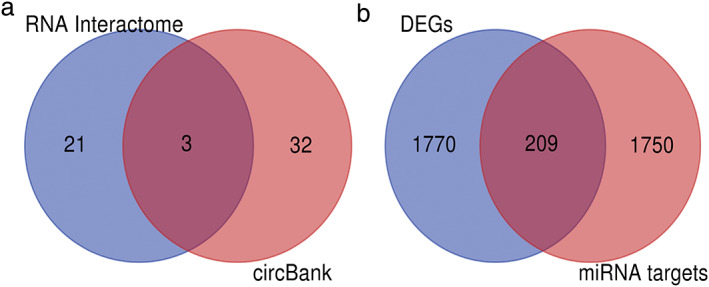
Identification of 209 potentially important genes in NSCLC. (a) Three sharing microRNAs. (b) Venn diagram for the intersections between DEGs and miRNA target genes

The circular RNA Interactome and circBank database was used to predict the potential target miRNAs of DECs. A total of 24 potential target miRNAs of hsa_circ_0043256 were obtained from circular RNA Interactome, and 35 potential target miRNAs were obtained from circBank. By merging these two screening results, three miRNAs (hsa‐miR‐1183‐5p, hsa‐miR‐1303‐5p, and hsa‐miR‐512‐5p) were obtained by Venn diagram (Figure 6(a)). miRWalk was used to predict the target mRNA of these three miRNAs. A total of 1959 potential target mRNAs were obtained.

**FIGURE 7:**
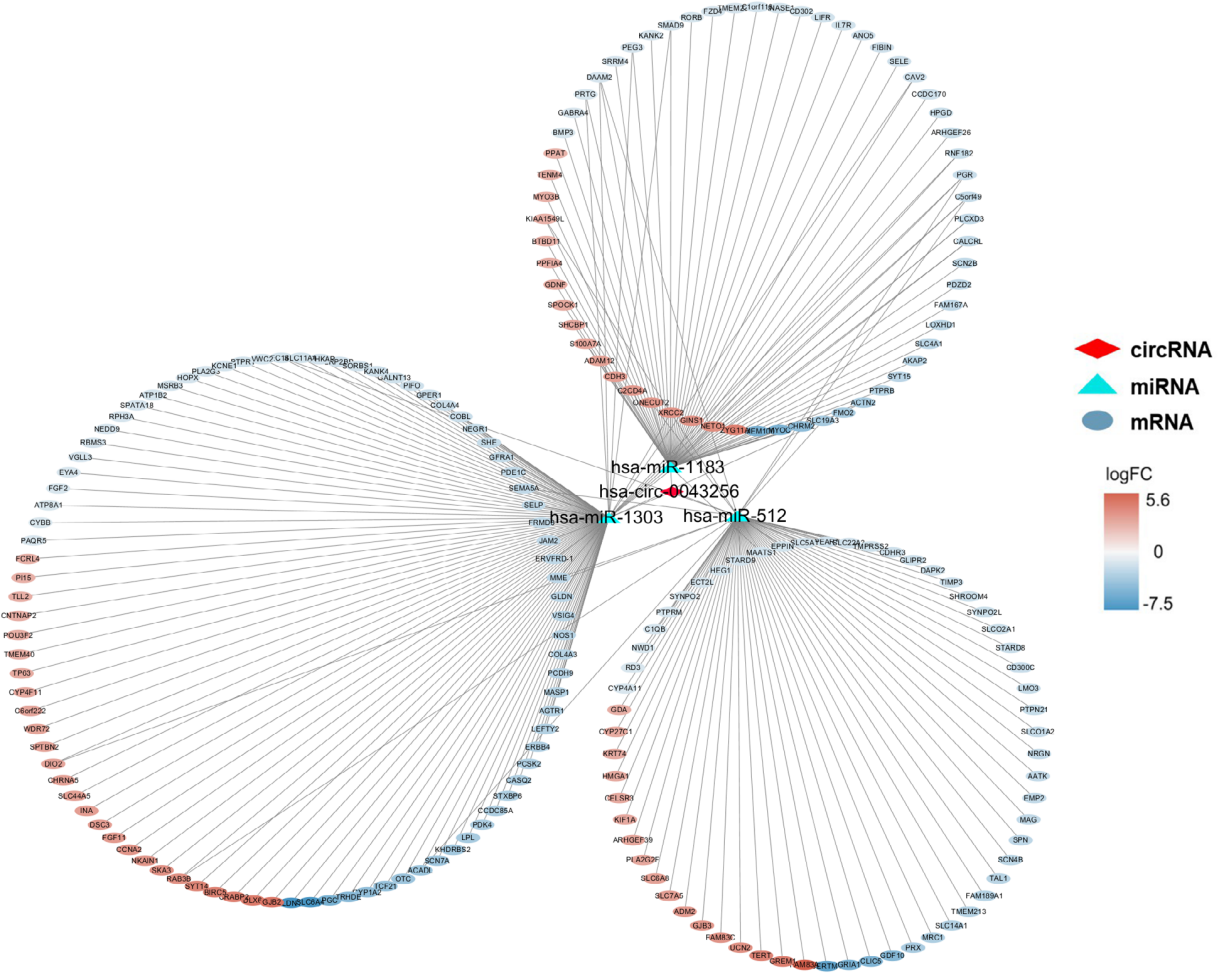
CircRNA–miRNA–mRNA regulatory network for hsa_circ_0043256 in NSCLC. The network consisted of one circRNAs (hsa_circ_0043256), three miRNAs (hsa‐miR‐1183‐5p, hsa‐miR‐1303‐5p and hsa‐miR‐512‐5p) and 209 genes

In addition, 1779 DEGs with 745 upregulated genes and 1034 downregulated mRNAs were identified from TCGA database for lung cancer tissues and normal lung tissues. A total of 209 overlapped genes were acquired to the construction of the miRNA–mRNA network using Venn diagram by intersecting the DEGs from TCGA and the targeted mRNAs of three miRNAs by miRWalk (Figure 6(b)). In an attempt to better understand the potential role of circRNAs, the network contained 1 circRNAs, 3 miRNAs, and 209 genes were obtained (Figure 7).

### Functional and pathway enrichment analysis

**FIGURE 8:**
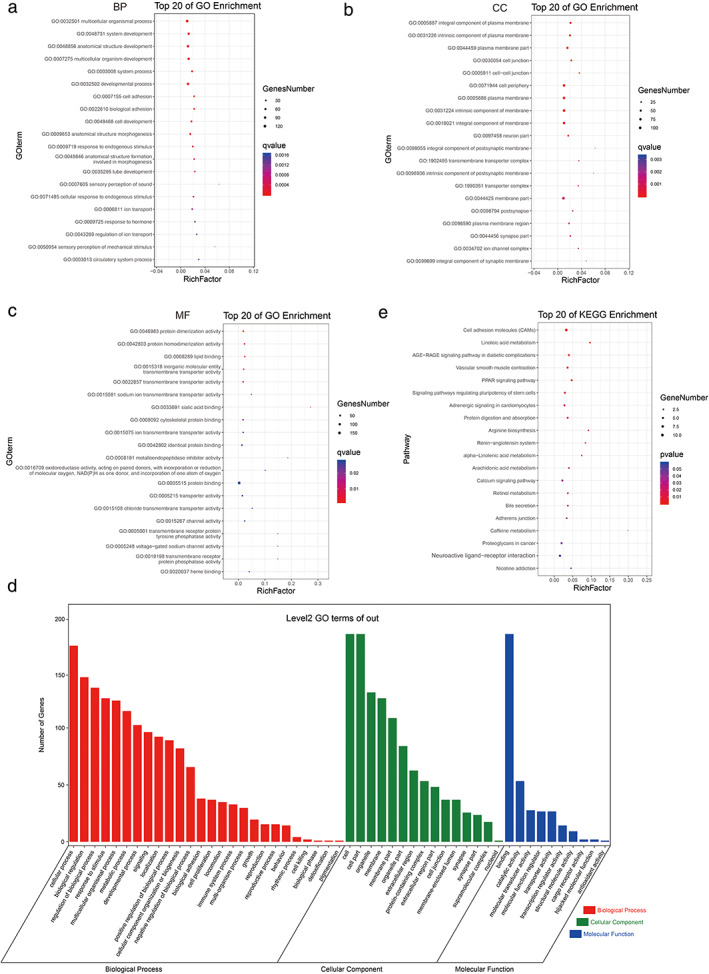
Functional analysis of 209 mRNAs in the network. (a)–(c) The dot plot of top 20 GO analysis. (d),(e) The dot plot of top 20 KEGG pathway analyses

To evaluate the function of the DEGs in the network, the 209 overlapped genes were used for GO and KEGG analysis. For GO analysis, with respect to the biological process (BP), the DEGs were enriched in GO:0007605 sensory perception of sound, GO:0050954 sensory perception of mechanical stimulus, and GO:0007155 cell adhesion. Regarding cellular components (CC), the top three enriched terms were GO:0099055 integral component of postsynaptic membrane, GO:0098936 intrinsic component of postsynaptic membrane, and GO:0005911 cell–cell junction. In terms of molecular function (MF), GO:0033691 sialic acid binding, GO:0008191 metalloendopeptidase inhibitor activity and GO:0016709 oxidoreductase activity, acting on paired donors, with incorporation or reduction of molecular oxygen, NAD(P)H as one donor, and incorporation of one atom of oxygen were the top three hits. KEGG pathway enrichment analysis revealed that several pathways were significantly enriched, including caffeine metabolism, linoleic acid metabolism, and arginine biosynthesis (Figure 8).

### Identification of hub genes from the PPI network

**FIGURE 9:**
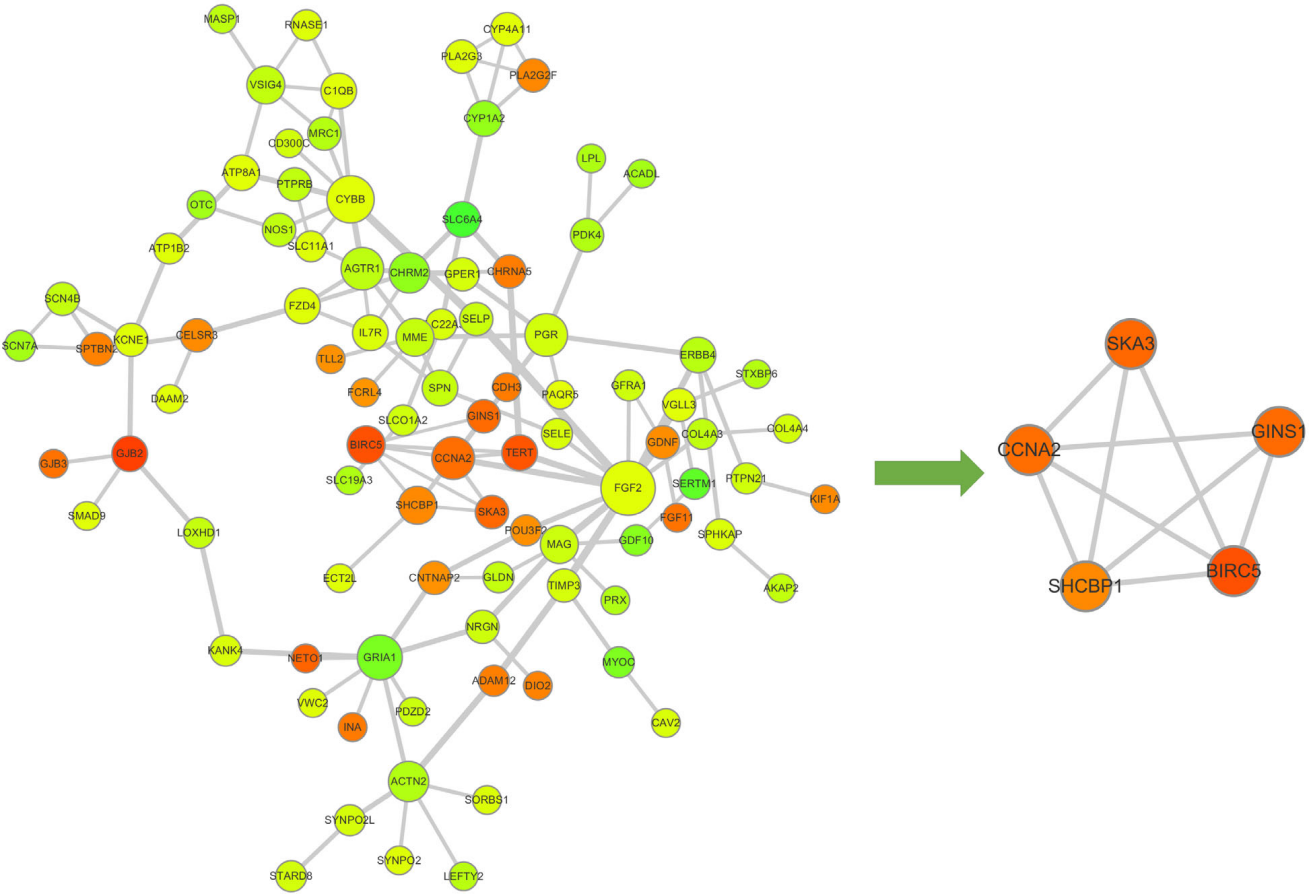
Identification of hub genes from the PPI network with the MCODE algorithm. The PPI network consisted of 139 nodes and 175 edges, and five hub genes that were extracted from the PPI network

Cytoscape software and the STRING database were used to construct the PPI network of the target genes of hsa_circ_0043256. A total of 139 nodes and 175 edges were mapped on the PPI network (Figure 9). The hub genes were collected by the MCODE algorithm. The top five hub genes were baculoviral IAP repeat containing 5 (BIRC5), SHC binding and spindle associated 1 (SHCBP1), cyclin A2 (CCNA2), spindle and kinetochore associated complex subunit 3 (SKA3), and GINS complex subunit 1 (GINS1).

### Relative mRNA expression of five hub genes by GEPIA database

**FIGURE 10:**
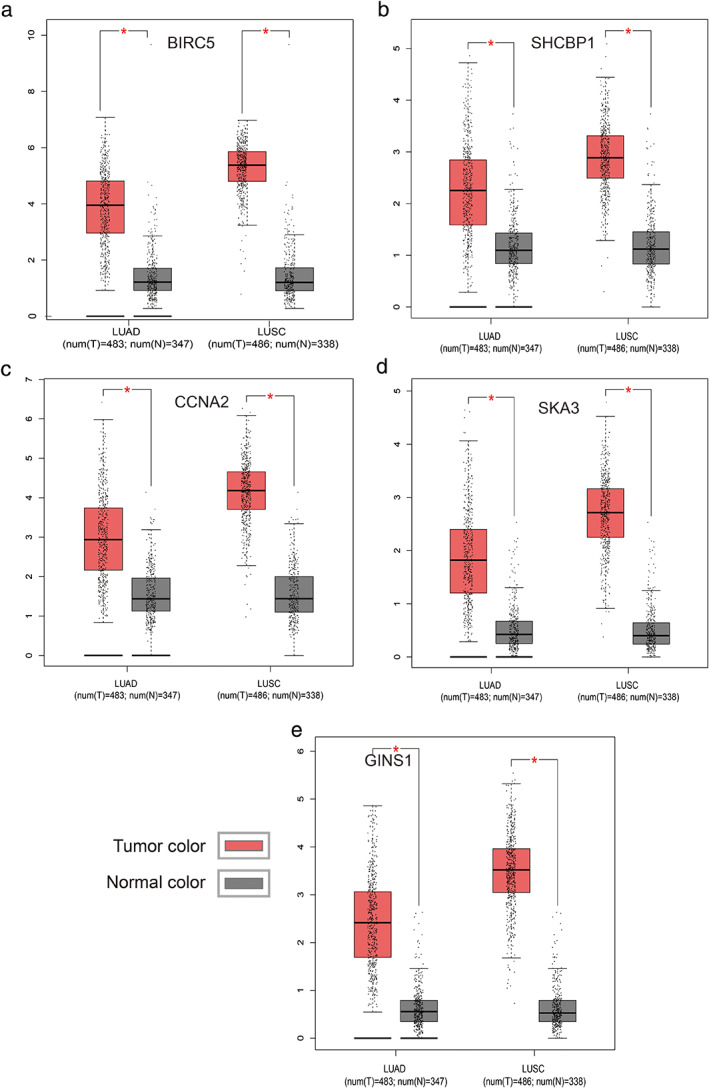
Validation of hub genes in the transcriptional level using the GEPIA database. (a) BIRC5. (b) SHCBP1. (c) CCNA2. (d) SKA3. (e) GINS1

The GEPIA database^11^ was used to analyze the mRNA expression levels of the five genes observed in lung cancer. As shown in Figure 10, the mRNA expression of BIRC5, SHCBP1, CCNA2, SKA3, and GINS1 in LUAD and lung squamous cell carcinoma (LUSC) tissues were all significantly higher than that in normal tissues.

### Relative expression of five hub genes in tissues by IHC and HPA database

**FIGURE 11:**
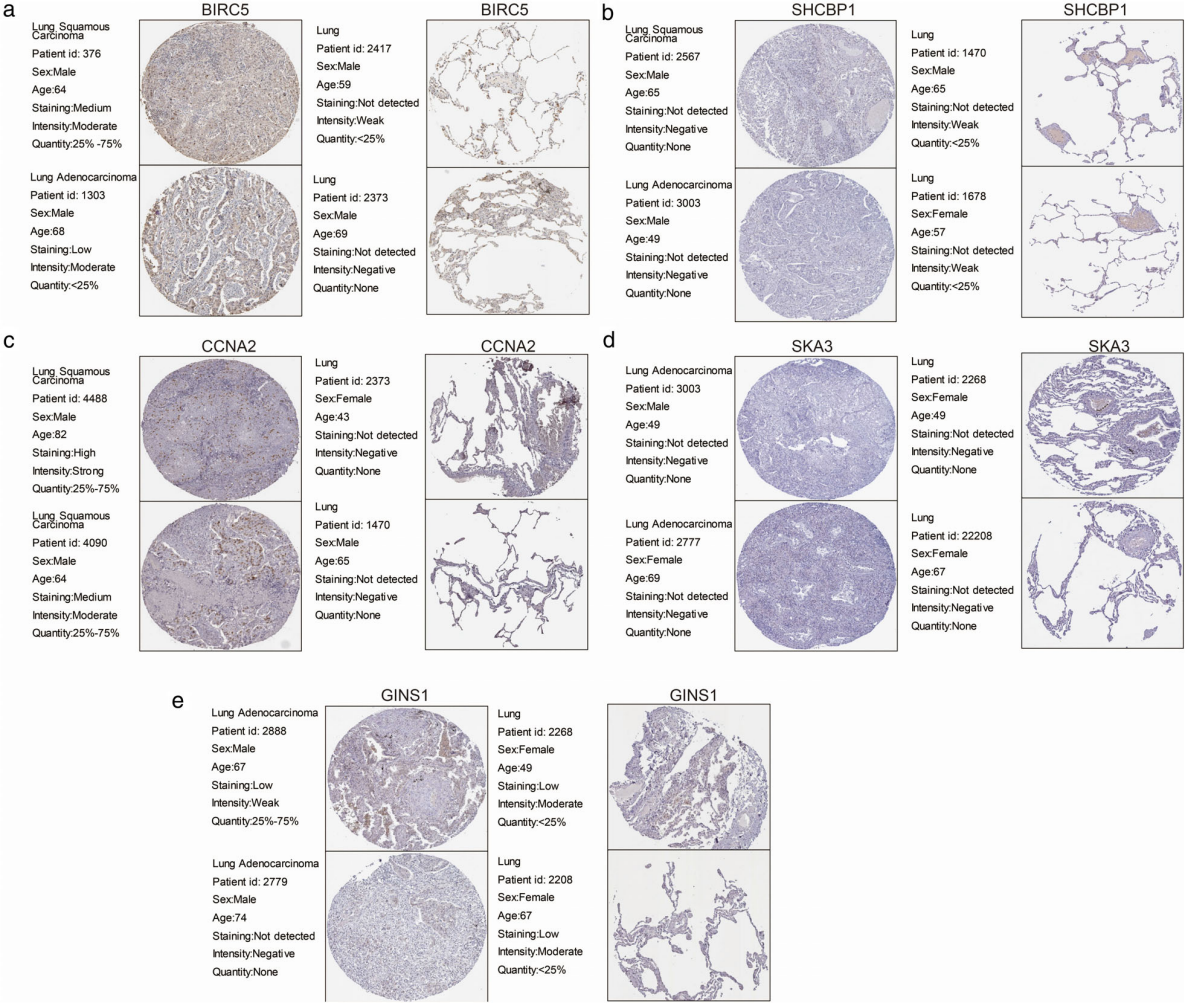
Validation of hub genes in the translational level using the HPA database. (a) BIRC5. (b) SHCBP1. (c) CCNA2. (d) SKA3. (e) GINS1

**FIGURE 12:**
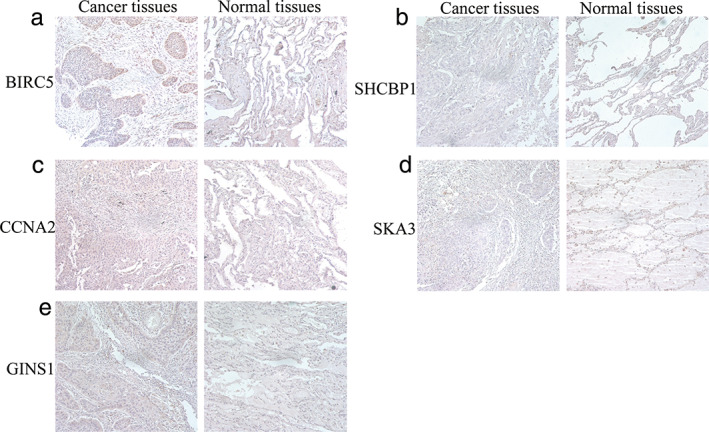
Validation of hub genes in the translational level using immunohistochemistry. (a) BIRC5. (b) SHCBP1. (c) CCNA2. (d) SKA3. (e) GINS1

Moreover, IHC staining obtained from the HPA database^12^ also clearly revealed that the expression levels of BIRC5 and CCNA2 in lung cancer tissues were much higher than those in the normal lung tissues. However, no significant differences were found in the remaining three hub genes (Figure 11). Similar results were confirmed by IHC assay in 12 patients (Figure 12).

### Survival analysis of five hub genes by KM plotter

**FIGURE 13:**
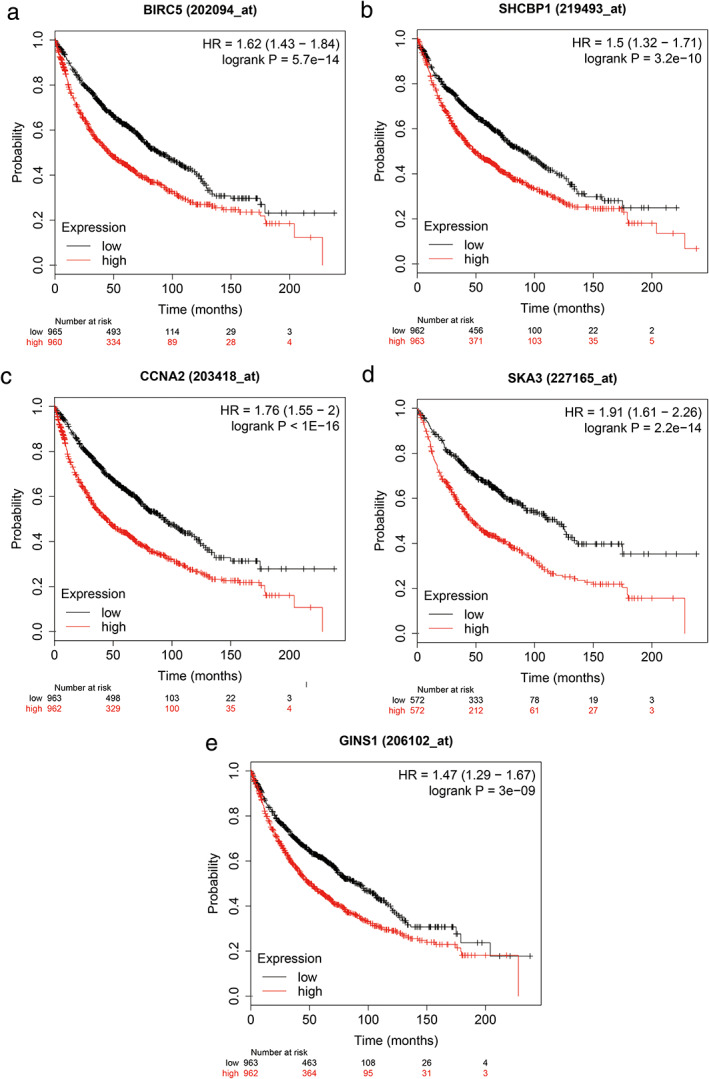
Overall survival for the five hub genes in NSCLC patients. (a) BIRC5 (HR = 1.62, *p* = 5.7e−14). (b) SHCBP1 (HR = 1.50, *p* = 3.2e−0). (c) CCNA2 (HR = 1.76, *p* < 1E−16). (d) SKA3 (HR = 1.91, *p* = 2.2e−14). (e) GINS1 (HR = 1.47, *p* = 3e−09)

The KM plotter was used to analysis the survival prognosis of all five hub genes. As shown in Figure 13, higher expression of BIRC5 (HR = 1.62, *p* = 5.7e−14), SHCBP1 (HR = 1.50, *p* = 3.2e−0), CCNA2 (HR = 1.76, *p* < 1E−16), SKA3 (HR = 1.91, *p* = 2.2e−14), and GINS1 (HR = 1.47, *p* = 3e−09) were significantly negatively correlated with poorer overall survival (Figure 13).

## DISCUSSION

In this study, we integrated three cohorts of profile datasets from different groups, used GO2R methods to analyze the DECs, and identified three commonly affected DECs (hsa_circ_0043256, hsa_circ_0029426, and hsa_circ_0049271) in the first step. Following RT‐qPCR validation of the three DECs, and hsa_circ_0043256 was found to be significantly downregulated in both lung cancer patient tissues and cell lines. Further, our results showed that hsa_circ_0043256 was capable of inhibiting cell proliferation and cell‐cycle progression of NSCLC cells in vitro. Therefore, hsa_circ_0043256 was selected for further analysis.

Numerous studies have shown that circRNA can bind to miRNAs and serve as ceRNAs and regulate gene expression.^6^, ^13^, ^14^, ^15^We next investigated the ability of hsa_circ_0043256 to bind miRNAs. Circinteractome and circBank database were used to explore the miRNA response elements (MREs) harbored by hsa_circ_0043256. We chose miRNAs predicted by both algorithms as the putative target miRNA for hsa_circ_0043256. Finally, three miRNAs (hsa‐miR‐1183‐5p, hsa‐miR‐1303‐5p, and hsa‐miR‐512‐5p) were selected. Increasing evidence has indicated that miRNAs may regulate gene expression by binding to the 3’ untranslated region (UTR) of some target genes. miRwalk was used to predict the potential target mRNAs of the three miRNAs. A total of 1959 potential target mRNAs were obtained. In addition, 1779 DEGs with 745 upregulated mRNAs and 1034 downregulated mRNAs were identified from the TCGA database between lung cancer and normal lung tissues. We chose the common 209 mRNAs of these two sections as the common DEGs for further analysis. Based on the results, we established the circRNA–miRNA–mRNA network, including one circRNA, three miRNAs, and 209 mRNAs, in an attempt to better understand the potential role of circRNAs.

To explore the potential mechanism of circRNAs on lung cancer, we performed functional analysis of the DEGs by GO and KEGG enrichment analysis. The GO analysis indicated that the DEGs were mainly involved in sensory perception of sound (GO:0007605), integral component of postsynaptic membrane (GO:0099055), and sialic acid binding immune response (GO:0033691). KEGG pathway analysis revealed that the DEGs were mainly associated with caffeine metabolism, linoleic acid metabolism, and arginine biosynthesis. Caffeine is a naturally occurring methylxanthine non‐selective adenosine receptor antagonist that can be found in many beverages like coffee and tea. Caffeine has been reported to have antitumor activity in animals and some cancer cell lines.^16^ Caffeine metabolism pathway has been reported to be the only significant metabolic pathway in predicting the survival outcome among NSCLC patients.^17^ It has been reported that caffeine may mediate DNA‐repair inhibition, and may be a higher risk factor for lung adenocarcinoma. This metabolism is mediated by genetics related to variations of specific genes. Although CYP1A2 is understood to be the main player, CYP2C8, CYP2C9, and CYP3A4 are also believed to participate in primary metabolism of caffeine.^18^, ^19^ Caffeine has been reported to enhance antitumor immune response through decreased expression of PD1 on infiltrated cytotoxic T lymphocytes.^20^ The linoleic acid (LA) metabolism pathway is first performed by Δ6‐desaturase, which converts LA into gamma‐linolenic acid (GLA).^21^, ^22^ Animals lack Δ6‐desaturase on their own, and acquire it through breast milk. Studies show that breast‐milk fed babies have higher concentrations of GLA.^23^ The metabolites of LA can mediate inflammation, which is understood to depend on FADS1 gene.^24^ The arginase pathway hydrolyzes arginine to urea and ornithine, which is then hydrolyzed by ornithine aminotransferase (RocD) and Δ‐pyrroline‐5‐carboxylate dehydrogenase (RocA), respectively, with the production of glutamate. Arginine metabolism consists of several cytosolic urea cycle enzymes, including argininosuccinate synthase (ASS), argininosuccinate lyase (ASL), and arginase and ornithine transcarbamylase (OTC), among which ASS is the key enzyme.^25^ ASS is a rate‐limiting biosynthetic enzyme for intracellular arginine synthesis in different cells. However, in some tumor cells like hepatocellular carcinoma, renal cell carcinoma, etc, it is reduced or even absent.^26^, ^27^ There is more evidence that indicates that arginine may be serving as a node for the regulation of immune responses.^28^ In signal transduction pathways in immune cells, arginine may be functioning, arginine may be as an enzyme substrate and also as a regulatory molecule.^29^ Because of the important role of arginine in cell proliferation and host immune responses, targeting the arginine metabolism pathway for arginine deprivation could be an effective treatment strategy for cancer patients.^27^

To further identify the key circRNAs that participate in the regulatory network, DEGs PPI network complex was developed and the most significant five hub genes, including BIRC5, SHCBP1, CCNA2, SKA3, and GINS1, were selected as the key in the PPI network. GEPIA and HPA databases were used to validate the five hub genes at transcriptional and translational levels. Results showed that the mRNA expression of BIRC5, SHCBP1, CCNA2, SKA3 and GINS1 in LUAD and LUSC tissues were all significantly higher than that in normal tissues, whereas only BIRC5 and CCNA2 protein expression in lung cancer tissues were much higher than those in the normal lung tissues. The KM plotter was used to show the correlation between hub genes and survival prognosis. Results showed that all hub genes were significantly related to survival analysis results.

BIRC5, also called survivin, is a member of the inhibitor of apoptosis (IAP) family.^30^, ^31^ BIRC5 is a direct target gene of the Wnt pathway and is upregulated by beta‐catenin.^32^ It has been shown that BIRC5 is a multifunctional protein that plays critical roles in several crucial cell processes such as apoptosis, cell proliferation, cell cycle, and chromosome movement.^30^ SHCBP1 is an important connexin on the SH2 domain of the SHC protein. It has been shown to play critical roles in cell proliferation, migration, adhesion, and cell‐cycle progression, especially in the carcinogenesis.^33^ Zou et al.^34^ found that SHCBP1 is remarkably upregulated in lung cancer, and overexpression of SHCBP1 promoted cisplatin induced apoptosis resistance, migration, and invasion. In addition, lung cancer patients with high SHCBP1 had poor prognosis. CCNA2 is the gene of cyclin‐A2 protein. Cyclin‐A2, one of the two types of cyclin A, is an essential regulator of the cell division cycle; it binds CDK2 during the S phase, and CDK1 during the transition from the G2 to M phase.^35^ Ruan et al.^36^ found that ectopic expression of CCNA2 accelerates NSCLC cell migration and invasion through integrin the αVβ3 signaling pathway in lung cancer cells, and suppression of CCNA2 expression inhibits metastasis of NSCLC cells. SKA3, a subunit of SKA1 complex, has been reported to be important in phosphorylation of Cdk1 in mitosis; it binds to Ndc80C and recruits the Ska complex to kinetochores.^37^ Previous studies have reported that SKA3 participates in cancer pathogenesis and progression.^38^, ^39^, ^40^ Hu et al.^38^ found that SKA3 is overexpressed in LUAD and its expression correlates with lymph node metastasis and poor prognosis. Further, reduced expression of SKA3 can significantly inhibit the metastasis of lung cancer cells. GINS1 is a subunit of the tetrameric complex termed GINS complex, which is essential for establishment of DNA replication forks. GINS has been suggested to be involved in DNA replication because of its binding to DNA replication protein.^41^ Several recent studies have suggested that GINS1 plays an important role in tumor growth.^42^, ^43^ For example, Zhang et al.^43^ found that PSF1 was overexpressed in lung cancer samples compared to adjacent non‐tumor samples, and knockdown of PSF1 was found to inhibit cell proliferation and caused cell‐cycle arrest of lung cancer cells in a p53‐independent manner.

In summary, in our study we constructed and analyzed a circRNA–miRNA–mRNA network of hsa_circ_0043256 based on the ceRNA theory via comprehensive bioinformatics analysis. This work may help to further explore the molecular mechanisms of lung cancer, because hsa_circ_0043256 may be a potential therapeutic target for lung cancer. Future studies are needed to explore the specific mechanisms of hsa_circ_0043256 and to better understand the role of these regulatory modules in the carcinogenesis of lung cancer.

## CONFLICT OF INTEREST

The authors declare no conflicts of interest.

## Supporting information


**Figure S1**. Differential expression of hsa_circ_0049271 and hsa_circ_0029426 in lung cancer and adjacent normal tissue. Scatter dot plots show the relative levels of hsa_circ_0049271 (A), and hsa_circ_0029426 (B).

**Table S1**. The clinical and pathological characteristics of 12 NSCLC patients
