# Supplementary figures and images for: Construction of a circular RNA–microRNA–messenger RNA regulatory network of hsa_circ_0043256 in lung cancer by integrated analysis
Source: Thorac Cancer. 2021 Nov 21;13(1):61–75. doi: 10.1111/1759-7714.14226 (PMC8720627; doi:10.1111/1759-7714.14226)

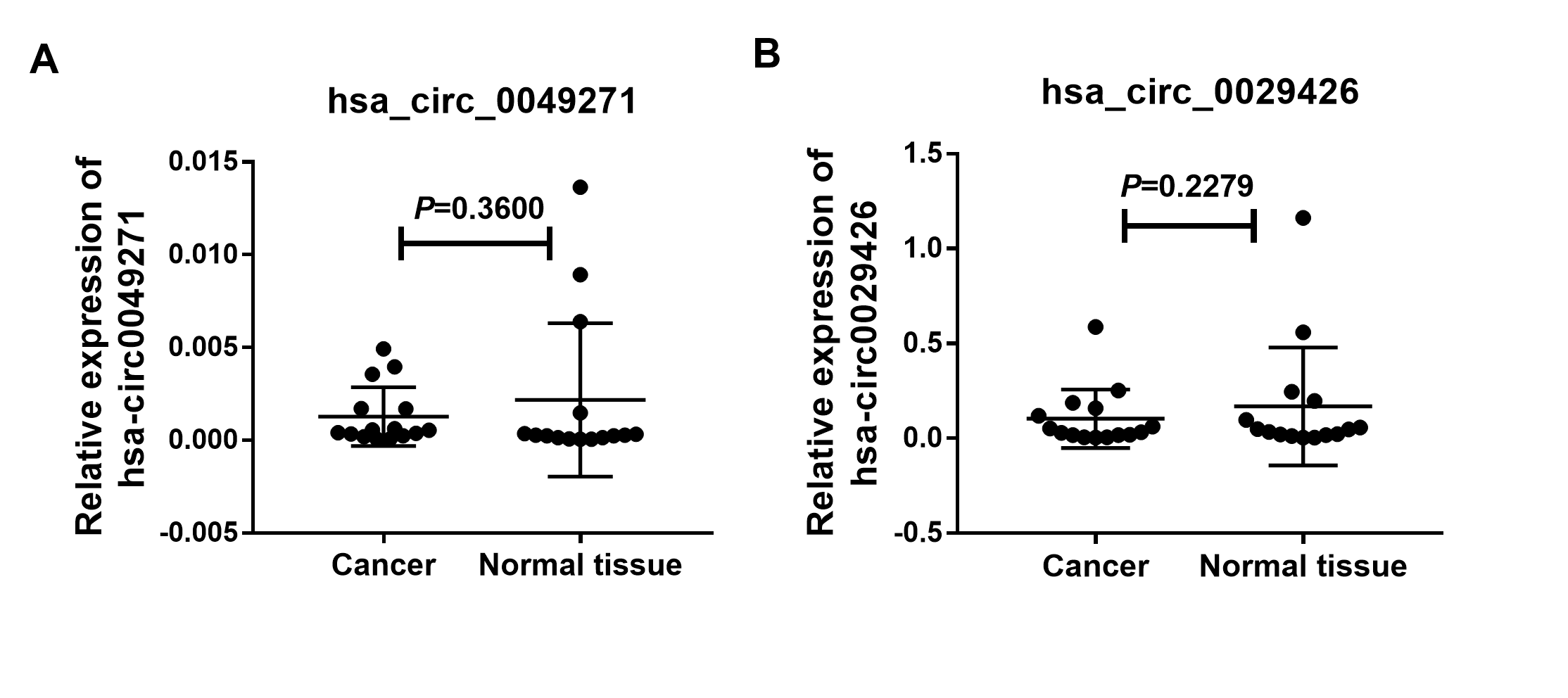

Supplement: Supplementary file 1 — Figure S1. Differential expression of hsa_circ_0049271 and hsa_circ_0029426 in lung cancer and adjacent normal tissue. Scatter dot plots show the relative levels of hsa_circ_0049271 (A), and hsa_circ_0029426 (B). [file TCA-13-61-s001.tif]
