# Supplementary material for: Construction of a circular RNA–microRNA–messenger RNA regulatory network of hsa_circ_0043256 in lung cancer by integrated analysis
Source: Thorac Cancer. 2021 Nov 21;13(1):61–75. doi: 10.1111/1759-7714.14226 (PMC8720627; doi:10.1111/1759-7714.14226)
Supplement: Supplementary file 2 — Table S1. The clinical and pathological characteristics of 12 NSCLC patients [file TCA-13-61-s002.doc]

**Suppl Table 1. The clinical and pathological characteristics of 12 NSCLC patients**

| ***Case number*** | ***Age*** | ***Gender*** | ***Smoking status*** | ***Histology***  ***（H&E）*** | ***pTNM*** |
| --- | --- | --- | --- | --- | --- |
| 1 | 65 | Female | Yes | AD | T2aN2M0 |
| 2 | 52 | Male | Non | SCC | T2aN2M0 |
| 3 | 56 | Female | Non | SCC | T3aN1M0 |
| 4 | 68 | Male | Yes | AD | T2aN0M0 |
| 5 | 61 | Male | Yes | AD | T2aN1M0 |
| 6 | 72 | Male | Yes | AD | T2aN1M1 |
| 7 | 65 | Female | Non | AD | T2aN0M0 |
| 8 | 72 | Female | Non | AD | T2aN0M0 |
| 9 | 73 | Male | Yes | AD | T2aN1M1 |
| 10 | 64 | Female | Non | AD | T2aN0M0 |
| 11 | 71 | Male | Non | AD | T2aN0M0 |
| 12 | 63 | Male | Non | AD | T2aN0M0 |

AD:adenocarcinoma;SCC:squamous carcinoma;
